# Investigating Environmental Matrices for Use in Avian Influenza Virus Surveillance—Surface Water, Sediments, and Avian Fecal Samples

**DOI:** 10.1128/spectrum.02664-22

**Published:** 2023-01-26

**Authors:** Ann Kathrin Ahrens, Hans-Christoph Selinka, Claudia Wylezich, Hubert Wonnemann, Ole Sindt, Hartmut H. Hellmer, Florian Pfaff, Dirk Höper, Thomas C. Mettenleiter, Martin Beer, Timm C. Harder

**Affiliations:** a Institute of Diagnostic Virology, Friedrich-Loeffler-Institut, Greifswald-Isle of Riems, Germany; b Section II 1.4 Microbiological Risks, German Environment Agency (UBA), Berlin, Germany; c State Laboratory of Schleswig-Holstein, Neumuenster, Germany; d Climate Sciences, Physical Oceanography of the Polar Seas, Alfred Wegener Institute, Bremerhaven, Germany; e Friedrich-Loeffler-Institut, Greifswald-Isle of Riems, Germany; University of Georgia

**Keywords:** avian influenza, surveillance, surface water, sediment, environment, filtration, feces

## Abstract

Surveillance of avian influenza viruses (AIV) in wild water bird populations is important for early warning to protect poultry from incursions of high-pathogenicity (HP) AIV. Access to individual water birds is difficult and restricted and limits sampling depth. Here, we focused on environmental samples such as surface water, sediments, and environmentally deposited fresh avian feces as matrices for AIV detection. Enrichment of viral particles by ultrafiltration of 10-L surface water samples using Rexeed-25-A devices was validated using a bacteriophage ϕ6 internal control system, and AIV detection was attempted using real-time RT-PCR and virus isolation. While validation runs suggested an average enrichment of about 60-fold, lower values of 10 to 15 were observed for field water samples. In total 25/36 (60%) of water samples and 18/36 (50%) of corresponding sediment samples tested AIV positive. Samples were obtained from shallow water bodies in habitats with large numbers of waterfowl during an HPAIV epizootic. Although AIV RNA was detected in a substantial percentage of samples virus isolation failed. Virus loads in samples often were too low to allow further sub- and pathotyping. Similar results were obtained with environmentally deposited avian feces. Moreover, the spectrum of viruses detected by these active surveillance methods did not fully mirror an ongoing HPAIV epizootic among waterfowl as detected by passive surveillance, which, in terms of sensitivity, remains unsurpassed.

**IMPORTANCE** Avian influenza viruses (AIV) have a wide host range in the avian metapopulation and, occasionally, transmission to humans also occurs. Surface water plays a particularly important role in the epidemiology of AIV, as the natural virus reservoir is found in aquatic wild birds. Environmental matrices comprising surface water, sediments, and avian fecal matter deposited in the environment were examined for their usefulness in AIV surveillance. Despite virus enrichment efforts, environmental samples regularly revealed very low virus loads, which hampered further sub- and pathotyping. Passive surveillance based on oral and cloacal swabs of diseased and dead wild birds remained unsurpassed with respect to sensitivity.

## INTRODUCTION

Avian influenza viruses (AIV) are influenza A viruses within the *Orthomyxoviridae* family. Their genome consists of eight segments of single-stranded RNA of negative orientation (1–3). Based on the two surface glycoproteins, hemagglutinin (HA) and neuraminidase (NA), which are embedded in a host cell-derived lipid envelope, AIV are grouped into 16 HA and 9 NA subtypes, whereas 2 further influenza A virus subtypes, H17N10 and H18N11, have been detected in bats only (4, 5).

AIV of HA subtypes H5 or H7 are further differentiated by their pathogenicity in chickens: high-pathogenicity (HP) AIV causes systemic infections leading to massive morbidity and mortality in chickens. HPAIV infection can cause high mortality also in other bird species. HPAIV infections in poultry have caused drastic economic losses worldwide, particularly after the emergence in Southeast Asia in 1996 and the subsequent global spread of the so-called goose/Guangdong lineage of H5 HPAIV (6). In contrast, infections with low-pathogenicity AIV (LPAIV) are usually focused on the gastrointestinal tract and induce a much milder course of infection. Frequently, they even remain asymptomatic, especially in populations of wild water birds of the orders *Anseriformes* (ducks, geese, swans) and *Charadriiformes* (gulls, terns, waders), which constitute the most important natural reservoirs of AIVs worldwide (7).

In contrast to mammalian influenza A virus infections, which essentially rely on respiratory infection and droplet-driven transmission, fecal-oral transmission plays a key role in the epidemiology of AIV (7–9). Many waterfowl species use shallow water habitats for foraging and resting. If infected birds congregate in such water bodies, AIVs could then be excreted in surface water through fecal contamination. This may turn such waters into a highly efficient source of infection (10). Once present in water, AIV can drift with currents, sink into sediments, or may otherwise be diluted and inactivated by various abiotic and biotic factors. Yet, the tenacity of AIV in surface water and in sediments is remarkably high for a virion whose infectivity depends on an intact lipid envelope (11–15). In addition, very few viral particles resuspended in surface water can be sufficient to start an infection, as recently shown by experimental infection studies in mallards (Anas platyrhynchos) (10). This highlights the putative importance of surface water as a transmission medium of AIV.

Surveillance of AIV in wild water bird populations is important for early warning purposes in protecting poultry populations from incursions of HPAIV (16–18). However, some waterbird species are highly endangered, and disturbance of their habitats should be avoided. This limits the sampling opportunities of the birds themselves. Instead, environmental matrices such as surface water that are potentially virus contaminated have been the focus of recent surveillance strategies (19, 20). The counterproductive effect of a dilution of the virus in larger water bodies or by currents needs to be considered when aiming at surface water as a surveillance target. Enrichment of viral particles therefore is an important step in increasing the sensitivity of detection. The spectrum of previously described methods focused on filtration (size or charge exclusion) often combined with ultracentrifugation or precipitation (21).

Charged filters have been found suitable for AIV enrichment from water (22). In general, negatively charged filters led to higher AIV recovery rates (22). Another method to concentrate AIV from smaller water volumes took advantage of formaldehyde-stabilized chicken erythrocytes for AIV binding; erythrocytes express sialic acid receptors utilized by influenza viruses to attach to permissive host cells (23). In addition, other methods such as ultracentrifugation, chromatography, and PEG precipitation were described for influenza A virus purification from cell culture supernatants but not from surface water (24–26). No general best-practice enrichment technology has been identified so far. The use of elution buffers to release particles from filter membranes grossly influenced downstream processing methods for the detection of AIV and poses a common dilemma: Detergents, alcohols, and aldehydes are expected to inactivate viral infectivity rapidly and would exclude virus isolation techniques in cell or egg cultures. However, using less harsh elution buffers might impair the recovery of viruses from filter membranes.

Here, we revisited previous attempts at AIV enrichment from surface water and sediments from various water bodies and types of surface water (fresh, brackish, salt). We combined and validated ultrafiltration techniques with several postfiltration enrichment steps and real-time RT-PCR detection. Environmental water, avian fecal samples, and wild bird carcasses were obtained from regions and during times of a high incidence of HPAIV H5N1 in the anseriform wild bird population. Despite our optimization attempts of active environmental surveillance, passive surveillance on diseased and dead birds remained superior in terms of early detection and measuring population infection trends with HPAIV.

## RESULTS

### Establishment and validation of bacteriophage ϕ6 as an internal surrogate marker of enveloped RNA viruses in surface water filtration and concentration experiments.

**FIG 1:**
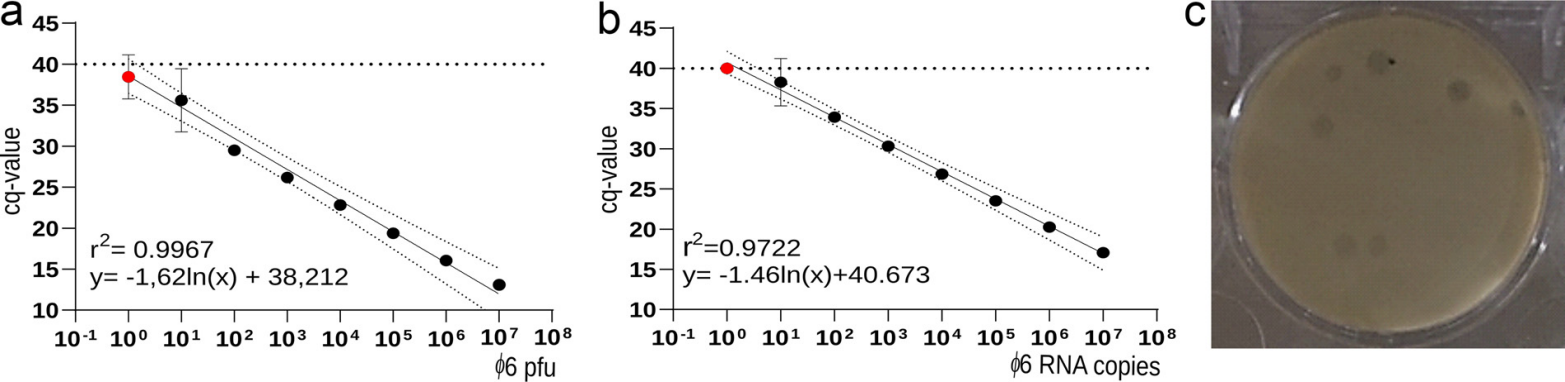
Performance characteristics of a TaqMan-based real-time RT-PCR (pair 3) for the detection of an M gene-specific fragment of bacteriophage ϕ6. RT-qPCR was assessed versus phage infectivity (PFU; a) and versus runoff RNA transcript copies of the target (b). Arithmetric averages and variation of triplicate experiments are shown. Red dots depict dilutions in which only 1 out of 3 replicates yielded *C_q_* values <40 defined as the threshold. (c) Typical results of the six-well plaque assay for bacteriophage ϕ6.

Here, we established an infectivity titration system in six-well soft agar plates on basis of plaque-forming units (PFU; Fig. 1), which was complemented by a sensitive, copy-based reverse transcription-quantitative PCR (RT-qPCR). Out of nine different primer pairs (pairs 1 to 9) and two (pairs 3 and 5) TaqMan probes (Table S1 in the supplemental material), the most sensitive one (pair 3) was selected. Figure 1 summarizes the performance characteristics of the optimum ϕ6 TaqMan RT-qPCR. As shown for other dsRNA viruses (27), an initial denaturation step of extracted RNA samples at 95°C for 5 min followed by a snap-cooling step improved PCR sensitivity by at least 1.5 log_10_ steps (not shown). The RT-qPCR tested with log_10_ dilutions of RNA runoff transcripts and RNA extracted from infectious phage particles revealed limits of detection at 10 RNA copies or PFU per reaction, respectively. On the basis of these results, 10 L of surface water was spiked with infectious ϕ6 phage particles as an internal control of enrichment and purification manipulations equivalent to 2.5 × 10^12^ RNA copies in total.

Downscaling the plaque assay used to determine ϕ6 infectivity titers from standard petri dishes to a six-well plate format had no effect on the test sensitivity (not shown).

### Comparison of filtration and concentration efficacy of Vivaflow and Rexeed ultrafiltration systems using spiked water samples.

Characteristics of two ultrafiltration devices, Rexeed and Vivaflow, were compared. In each case, 10 L of Baltic Sea surface water, taken during the summer months of 2020 from the harbor basin at the FLI located on a shallow outlet on the southern coast of the Baltic Sea (54°8’40.428’’ N; 13° 29’ 0.021’’E), was spiked with infectious ϕ6 phage particles equivalent to 2.5 × 10^12^ RNA copies. In addition, 100 μL of MDCK-II-grown supernatant containing AIV of subtype H9N2 at a titer of 5.62 × 10^7^ TCID_50_ mL^−1^ was added as a positive target control in validation runs. After addition of the two controls, the 10-L samples were stirred for 30 min at room temperature to achieve homogenous particle distribution.

For standardization, both filtration systems were tested repeatedly (Vivaflow, *n* = 2; Rexeed, *n* = 12). The Rexeed columns were tested with two different elution buffers: 0.2× PBS with (*n* = 8) or without 0.001% Tween 80 detergent (*n* = 4). For Rexeed, the maximal flow rate reached 268.46 mL/min (±15.09 mL/min) versus 45.2 mL/min (±6.0 mL/min) for Vivaflow. Thus, the Rexeed system had processed the 10-L samples on average in 40 min; elution adds another 10 min completing the full cycle in less than 1 h. Aliquots of the original 10-L samples as well as the eluate (Rexeed) and the concentrate (Vivaflow), respectively, each of 150 mL, were tested for viral loads by RT-qPCR and for infectivity by virus isolation in embryonated chicken eggs (AIV H9N2, two to four samples per method) or ϕ6-plaque assay, respectively (Table 1; Table S2).

**TABLE 1:** Validation of two ultrafiltration devices (Vivaflow, Rexeed) for enrichment of enveloped RNA virions (bacteriophage ϕ6, avian influenza virus H9N2) from surface water samples^*a*^

Target	Parameter	Vivaflow (*n* = 2)	Rexeed PBS (*n* = 8)	Rexeed PBST (*n* = 4)
ϕ6				
10 L	*C_q_*	31.98	29.81	31.72
150 mL	*C_q_*	27.80	23.49	25.66
Delta *C_q_*	*C_q_*	4.18 (±0.51)	6.32 (±1.05)	6.06 (±0.35)
10 L	RNA copies	1.91 × 10^4^	9,58 × 10^4^	2.43 × 10^4^
150 mL	RNA copies	4.73 × 10^5^	1.44 × 10^7^	2.74 × 10^6^
Factor	*n*	2.99 × 10^1^ (±1.1 × 10^1^)	1.93 × 10^2^ (±1 × 10^2^)	1.27 × 10^2^ (±3.37 × 10^1^)
10 L	PFU	2.09 × 10^5^	4.05 × 10^7^	9.0 × 10^5^
150 mL	PFU	9.07 × 10^2^	8.29 × 10^8^	1.31 × 10^7^
Factor	*n*	1.26 × 10^−2^ (1.26 × 10^−2^)	4.64 × 10^1^ (±7.2 × 10^1^)	1.49 × 10^1^ (±4.1 × 10^0^)
H9N2				
10 L	*C_q_*	30.91	30,99	31.18
150 mL	*C_q_*	27.62	25.54	26.08
Delta	*C_q_*	3.29 (±0.13)	5.45 (±0.65)	5.11 (±1.83)

a Values in parenthesis indicate the standard deviation. Samples were spiked with known amounts of ϕ6 and AIV isolate H9N2. Target detection was by specific RT-qPCRs (*C_q_* values and, for ϕ6, corresponding RNA copies are shown) and by virus isolation (plaque assay for ϕ6; qualitative isolation in ECE for H9N2). Elution buffers were with (PBST) or without (PBS) supplementation of Tween 80.

As summarized in Table 1, for both spike systems, bacteriophage ϕ6 and AIV H9N2, a basic value in RT-qPCRs of around quantification cycle (*C_q_*) 30 was achieved when analyzing aliquots of the original 10-L samples (details for each run separate are depicted in Table S2). Each of the ultrafiltration devices yielded an enrichment of viral RNA. Independent of the elution buffer, the Rexeed system led to a higher concentration than the Vivaflow system when comparing delta *C_q_* values (ϕ6 and H9N2) and enrichment factors of RNA copies (ϕ6). With respect to the different elution buffers of the Rexeed system, marginally higher enrichment factors were obtained by the 0.2× PBS buffer without Tween although yields varied considerably between different runs compared to the Tween buffer protocol. Interestingly, elution with either 0.2× PBS or 0.2× PBS+ 0.001% Tween 80 yielded infectious virus both with ϕ6 and H9N2. Tween-containing elution buffer led to reduced recovery rates for ϕ6, although infectious H9N2 was recovered from both elution buffer types (Table 1). In the Vivaflow system, ϕ6 infectivity was lost to a great extent while qualitative virus isolation for H9N2 still yielded positive results.

Based on these validation data, as well as on technical considerations, including ease of handling and reduction of time and costs (Table S3), the Rexeed system using PBS elution buffer without Tween supplement was found superior and used in all examination of field samples. The Vivaflow system was excluded also on basis of technical terms due to the frequent blocking of filters.

### Attempts at postfiltration enrichment increasing AIV RNA recovery.

**FIG 2:**
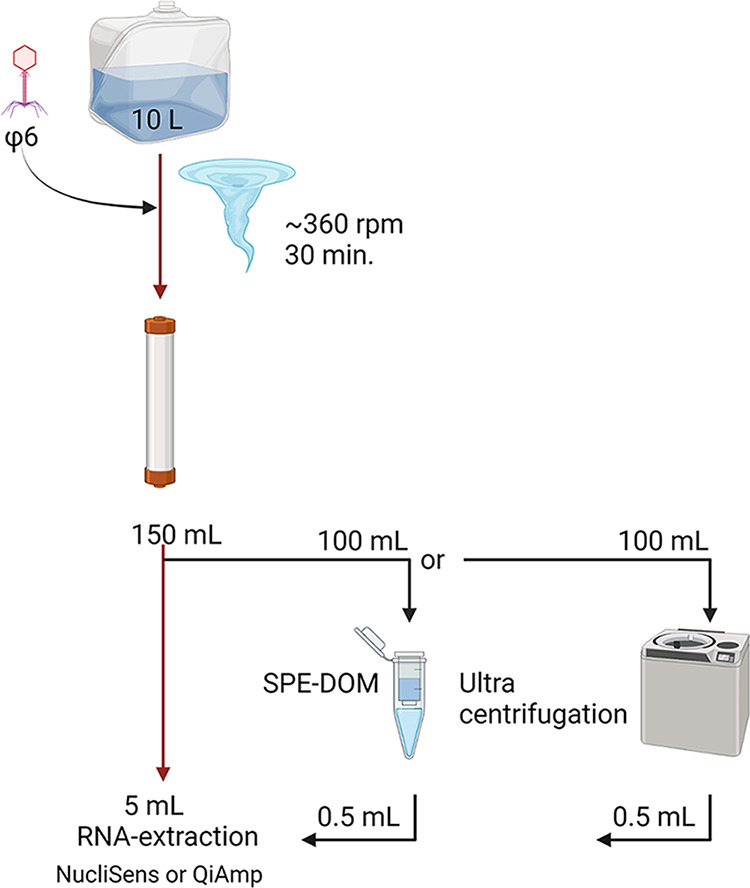
Flow diagram for influenza A virus and bacteriophage ϕ6 particle enrichment from surface water samples using Rexeed columns and postfiltration measures (ultrafiltration and centrifugation), tested during validation (black arrows) and finally applied (red arrows).

We attempted to further concentrate nucleic acids from the 150 mL of Rexeed filtration eluates comparing solid-phase extraction of dissolved organic matter filtration (*n* = 3), as described in reference 28, and particle-associated ultrafiltration (*n* = 3) using eluate aliquots of 100 mL each. With SPE-DOM, all RNA and infectivity present in the eluate were consistently lost and seemed to be fully adsorbed to the filter material (not shown). Ultrafiltration, in contrast, led to the recovery of particles testing positive by RT-qPCR but no quantitative gain in recovery rates could be verified compared to Rexeed filtration alone (not shown). Based on these results, field water samples were subsequently processed using solely the Rexeed system without applying any postfiltration enrichment procedures (compare Fig. 2).

### Examination of surface water and sediment samples from the field.

**FIG 3:**
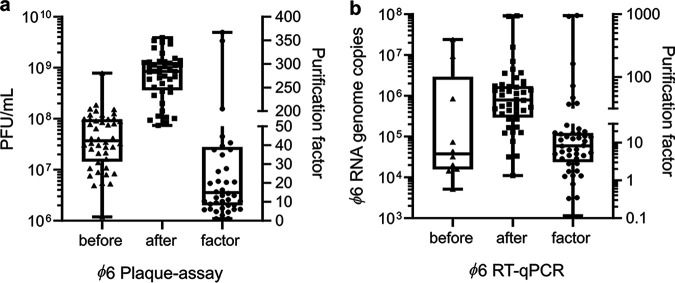
Recovery rates of bacteriophage ϕ6 spiked into field samples of surface water as a measure of enrichment efficacy by Rexeed-assisted ultrafiltration. Infectivity (a) was measured by plaque formation assay (PFU/mL; left scale) and by RT-qPCR (b; RNA copies; left scale) before and after filtration. Corresponding enrichment factors were calculated (a and b; right scales).

**FIG 4:**
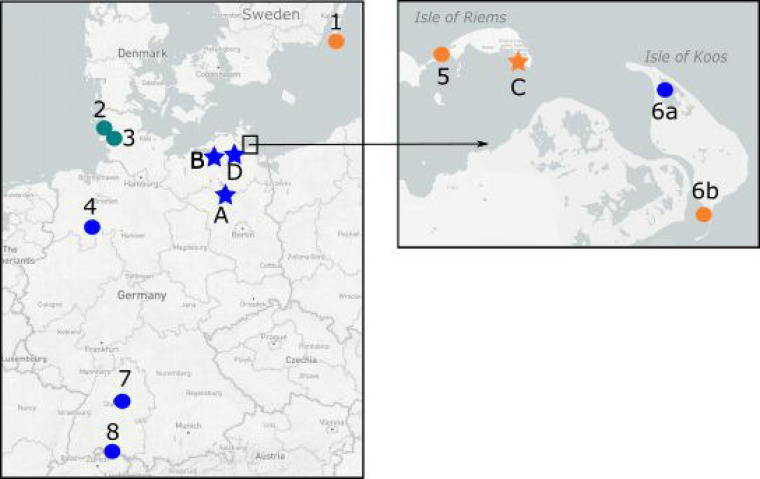
Geographic locations of sampling sites for surface water and corresponding sediments. Dots, scheduled samples taken on a routine base; stars, HPAI outbreak samples. Color represents water sample type: Blue, fresh; ochre, brackish; green, salt.

44 surface water samples were examined for the presence of AIV-specific RNA following Rexeed-assisted ultrafiltration. All field water samples were spiked with ϕ6 phage particles as an internal marker of enrichment efficacy. By ϕ6-specific RT-qPCR and plaque formation assay, recovery rates were controlled and shown to yield variable enrichment efficacies, which on average were lower than validation runs (enrichment factor 10 to 15 versus 60; Fig. 3). This indicates a certain robustness of the Rexeed-assisted ultrafiltration procedures with water samples of different origin (fresh, salt and brackish water). However, the higher content of floating particles/sediment in the majority of samples might have reduced recovery rates. As a positive control for the detection of AIV, a sample was obtained from a small water pool of 100 L used by 10 mallards during an HPAIV H5N8 infection experiment (10). This sample, expectedly, yielded a high virus load (*C_q_* 26.47) after ultrafiltration, sufficient for full sub- and pathotyping (Table 2, sample 57). True field samples were obtained from various locations in Sweden (*n* = 1; bird trap on the island of Øland, Baltic Sea, Sweden), Germany (*n* = 33), and the Antarctic Weddell Sea (*n* = 5) (Table 2, Fig. 4, and Fig. S1). Shallow water bodies on the island of Koos, Germany, at the southern coast of the Baltic Sea, were sampled repeatedly over a period of 19 weeks. Five additional samples were taken from open water bodies on holdings of poultry (*n* = 2) or kept birds (*n* = 2), which experienced acute HPAIV outbreaks during a period of a high incidence of HPAIV infections in wild birds in the respective regions in northern Germany (Fig. 4, stars).

**TABLE 2:** Analyses for presence of avian influenza viruses of Rexeed-filtrated surface water samples and corresponding sediment samples from different sources^*a*^

Sampling site^*b*^	Sample no. and type^*c*^	Water				Sediment		
		AI RT-qPCR before Rexeed	AI RT-qPCR after Rexeed^*d*^	Subtype	Pathotype	AI RT-qPCR	Subtype	Pathotype
1	1	Neg	37.08	Not H5/H7	n.e.	32.07	H2, H4, H5, H6, N3, N9	Not typable
6a	2	35.87	33.70*	H6, N2, not H5/H7	n.e.	Neg	n.e.	n.e.
6b	3	Neg	36.68*	Not H5/H7	n.e.	Neg	n.e.	n.e.
3	5	36.06	35.55	H5	Neg	34.92	H5	Not typable
8	6	Neg	35.02*	Not H5/H7	n.e.	Neg	n.e.	n.e.
6b	7	n.e.	Neg	n.e.	n.e.	40.19	H5	n.e.
2	12	38.02	37.68	Not H5/H7	n.e.	35.32	H5, H6, N3, N9	H5-LP, H5-2.3.4.4b
6a	13	n.e.	Neg	n.e.	n.e.	37.36*	Not H5/H7	n.e.
6b	14	Neg	37.51	H5	Not typable	34.15*	Not H5/H7	n.e.
8	15	n.e.	Neg	n.e.	n.e.	34.53*	Not H5/H7	n.e.
7	17	n.e.	35.76*	Not H5/H7	n.e.	35.13*	Not H5/H7	n.e.
4	18	Neg	36.56*	Not H5/H7	n.e.	Neg	n.e.	n.e.
6b	19	Neg	35.70*	Not H5/H7	n.e.	37.37*	Not H5/H7	n.e.
6a	20	n.e.	39.67	Not H5/H7	n.e.	Neg	n.e.	n.e.
6b	22	n.e.	Neg	n.e.	n.e.	Neg	n.e.	n.e.
6a	23	n.e.	Neg	n.e.	n.e.	Neg	n.e.	n.e.
5	25	Neg	36.03	Not H5/H7	n.e.	Neg.	n.e.	n.e.
8	26	Neg	35.45	Not H5/H7	n.e.	37.05*	Not H5/H7	n.e.
7	27	n.e.	37.79	Not H5/H7	n.e.	36.54	Not H5/H7	n.e.
6b	28	n.e.	Neg	n.e.	n.e.	35.77	Not H5/H7	n.e.
6a	29	n.e.	Neg	n.e.	n.e.	Neg	n.e.	n.e.
6b	30	n.e.	Neg	n.e.	n.e.	37.15	H5, H7	Not typable
6a	31	n.e.	Neg	n.e.	n.e.	38.12*	Not H5/H7	n.e.
7	32	n.e.	Neg	n.e.	n.e.	35.04	Not H5/H7	n.e.
4	33	n.e.	Neg	n.e.	n.e.	Neg	n.e.	n.e.
6a	34	Neg	38.51	Not H5/H7	n.e.	Neg	n.e.	n.e.
6b	35	Neg	35.57*	Not H5/H7	n.e.	Neg	n.e.	n.e.
6b	44	Neg	36.73	Not H5/H7	n.e.	Neg	n.e.	n.e.
6a	45	Neg	36.37	Not H5/H7	n.e.	Neg	n.e.	n.e.
8	46	38.23*	35.34	Not H5/H7	n.e.	33.92	Not H5/H7	n.e.
6a	47	Neg	36.27	Not H5/H7	n.e.	Neg	n.e.	n.e.
6b	48	Neg	39.64	Not H5/H7	n.e.	Neg	n.e.	n.e.
7	49	Neg	35.44	Not H5/H7	n.e.	36.93	Not H5/H7	n.e.
8	50	Neg	36.37	Not H5/H7	n.e.	Neg	n.e.	n.e.
A	4	Neg	36.98*	H5	Not typable	Neg	n.e.	n.e.
B	16	Neg	37.82	Not H5/H7	n.e.	34.62*	Not H5/H7	n.e.
C	21	Neg	39.1	Not H5/H7	n.e.			
D	24	Neg	34.84*	Not H5/H7	n.e.			
M, G	57	32.79*	26.24	H5, N8	HP-2.3.4.4b			

a Neg, *C_q_* ≥40; n.e., not examined; M, sample from mallard infection experiment (10).

b See Table 4 and Fig. 4 for numbering of sampling site and type of water sample.

c Water sample type is indicated by no shading (fresh), light-gray shading (brackish), or dark-gray shading (salt).

d Asterisks represent 1:10 dilution.

In 27 out of 44 true field water samples (61%), AIV-specific RNA could be detected. The *C_q_* values ranged from 33.7 to 39.67 signaling low virus loads. RNA isolated from nine sample eluates revealed PCR inhibitory effects and had to be retested at a 1:10 dilution (Table 2, marked by an asterisk). From these samples, virus loads up to 3.3 *C_q_* values higher are to be expected lowering the range to arithmetically *C_q_* 30.4 for sample 2. For four samples, subtyping by RT-qPCR was successful. This included sample 2 showing the highest virus load that contained AIV of subtype H6N2. Three further samples generated a positive signal for subtype H5, but no corresponding NA subtype could be assigned. Likewise, pathotyping by RT-qPCR did not produce valid results due to the low virus loads. Interestingly, all five samples from HPAIV outbreak holdings tested positive for AIV of which two were also subtyped as H5. None of the ocean water samples collected in the Antarctic Weddell Sea yielded a positive signal (data not shown). Virus isolation was unsuccessful.

For a subset of 36 water samples, corresponding sediments were available. 18 sediment samples (50%) tested positive in the generic RT-qPCR with average *C_q_* values of 35.9 (range, 32.07 to 38.04). For the matched surface water samples, 25 out of 36 (69%) tested AI positive by RT-qPCR (36.6, range, 33.07 to 38.78), but no significant differences in average *C_q_* values were evident (Table 2). Fourteen samples were congruently positive, and four were negative in water and sediment extracts. Twelve samples tested positive in water only and seven were positive in sediments only. For each type, eight samples revealed PCR inhibitory factors. Subtyping was possible for four and five of the matched water and sediment samples, respectively. The range of subtypes detected appeared to be broader for the sediment type (Table 2). Six different HA (2, 4, 5, 6, 7, and 9) and three different NA (3, 6, and 9) were detected in sediments whereas in water samples HA5 and 6 and N2 were distinguished. Pathotyping was possible in a single sediment sample only. As with the water samples, no AIV was isolated from any of the sediment samples.

### Metagenome sequencing of a field water sample.

RNA extracted from water sample 5 was subjected to a metagenome sequencing approach because of its comparatively high virus load for H5 with a *C_q_* value of 33.55 (Table 2). The data set obtained from the generic sequencing workflow yielded 5,749,075 reads in total. The generic approach of the metagenome analysis of the sample primarily showed a broad spectrum of viruses (*n* = 2,877), bacteria (*n* = 1,623,636), archaea (*n* = 9,807), and eukaryotes (*n* = 4,020,940) present in the sample. Members of a total of 29 different virus families could be detected. The internal marker bacteriophage ϕ6 was highly represented (1,039 reads, 0.02%) likely due to the specific spiking of the sample. However, the generic approach failed to generate any AIV-specific reads despite the positive results of the generic and H5-specific RT-qPCRs. Using a baits-based enrichment approach, however, 288 AIV reads (0.08%) were detected in the sequencing data set of 359,114 reads in total. A blastn analysis (https://blast.ncbi.nlm.nih.gov/Blast.cgi) assigned all of those reads to subtype H5N8. A full AI virus genome, however, could not be assembled from those reads. Likewise, pathotyping based on HA sequences was not possible. Sequences were submitted to NCBI and are available under accession numbers OP615145–OP615148.

### Examination of environmental fecal wild bird samples.

Environmental avian fecal samples were tested as another leg of AIV wild bird surveillance. Here, a region and time were chosen for a high incidence of HPAIV H5 clade 2.3.4.4b among populations of anseriform wild birds along the Wadden Sea coast of the German Federal State of Schleswig-Holstein from November 2020 to April 2022. Fecal samples were collected by ornithologically experienced rangers of the Wadden Sea national park. To correlate fecal samples and corresponding bird species, sampling was restricted to sites where large flocks of barnacle (Branta leucopsis) and brent geese (Branta bernicla) or Eurasian wigeons (Mareca penelope) were spotted immediately before sampling. At the same time and region, but not necessarily at the same spots, carcasses of the three species were retrieved and oropharyngeally and cloacally swabbed. Results of AIV RT-qPCRs performed on fecal samples and swabs are summarized in Table 3. A considerable proportion of fecal samples (52%) but not of swabs revealed the presence of PCR inhibitors and had to be reexamined in a 1:10 dilution. A total of 2,120 fecal samples and 115 carcasses were finally examined. From that total, 83.8% of all carcasses tested positive for AIV (*C_q_* 26. 4 ± 5.2), and 79.4% of the carcasses tested positive for HPAIV H5 of clade 2.3.4.4b with, on average, high viral loads (*C_q_* 24.8 ± 5.2). A total of 89 fecal samples (4.2%) harbored AIV-specific RNA with viral loads ranging from *C_q_* 31.1 to 39.3; 26 samples could be subtyped revealing a broad spectrum of subtypes as listed in Table 3. Among them, viruses of subtypes H5 (*n* = 13) and H7 (*n* = 1) were also present. Pathotyping was successful for six fecal samples identifying HPAIV H5 of clade 2.3.4.4b in five and LPAIV H5 in one sample.

**TABLE 3:** Results of active (environmental avian fecal samples) and passive surveillance (swabs of avian carcasses) for avian influenza viruses in three anseriform bird species during epizootics of HPAIV H5 along the Wadden Sea coast of the German Federal State of Schleswig-Holstein^*a*^

Species	Time	Environmental fecal samples			Swabs (carcasses)	
		Total/AI^*b*^	Subtype	Pathotype	Total/AI^*b*^	Sub- and pathotype
*Branta leucopsis*	12/20	20/0			8/6	HP H5Nx (2), HP H5N5 (1), HP H5N8 (4)
	01/21	390/4	H9N2 (3), H5N2 (1)			
	02/21	390/24	H5Nx (2), H5N6 (1), Not H5/H7 (17), H7 (1), H5 (1), n.e. (2)		6/6	HP H5N8 (6)
	03/21	580/33	Not H5/H7 (29), H5 (1); [H9, N2, H5, N3] (1),[H9, N2, H10, H5 (1)]	H5-LP (1), 2.3.4.4b (1)	5/5	HP H5N1 (1), HP H5N8 (4)
	11/21	125/3		H5-HP (1), 2.3.4.4b	6/6	HP H5N1 (6)
	12/21	100/2			5/4	HP H5N1 (4)
	01/22	25/0			10/9	HP H5N1 (9)
	02/22	50/2	Not H5/H7 (2)		9/8	HP H5N1 (5)
	03/22	25/3	H5 (2)	2.3.4.4b (1)	11/9	HP H5N1 (9)
Sum *Branta leucopsis*		1,705/71			65/56	53
*Branta bernicla*	01/21	20/2	H3N8 (2)			
	02/21	20/2	Not H5/H7 (1), n.e. (1)			
	03/22	75/4	Not H5/H7 (2), N1 (1), N8 (1)	H5-HP-2.3.4.4b (2)	1/1	HP H5N1 (1)
Sum *Branta bernicla*		115/8			1/1	1
*Mareca penelope*	12/20	20/3	H4N6 (2), n.i. (1)		1/0	
	01/21	85/0				
	02/21	25/0				
	03/21	70/4	Not H5/H7 (3), H5 (1)			
	11/21	50/2				
	12/21	50/1				
	03/22				2/0	
Sum *Mareca penelope*		300/10			3/0	
Sum all		2,120/89			68/57	54

a Over the complete sampling time (November 2020 until April 2022), 12% of the suspected passive surveillance sampled were AIV negative and 15% were negative for HPAIV. n.i., not identifiable; n.e., not examined.

b Positive for AIV RNA by generic RT-qPCR.

## DISCUSSION

HPAIV of the gs/GD lineage remains a threat to poultry populations worldwide. In Europe, seasonal reoccurrence during the winter months in migratory wild bird populations and subsequent spread to and among poultry holdings have been observed (8, 29). In Germany, since 2016/2017, there is a trend toward an increasing number of cases in wild birds and poultry (30). In parallel, a tendency for the year-round presence of HPAIV in the wild bird population in northern Europe has been reported (31). As a response to the year-round high incursion pressure of HPAIV, enhanced biosecurity measures for poultry holdings included restricted outdoor rearing and trading activities. Improved early warning for HPAIV incursion pressure is expected to limit the period of restrictive biosecurity measures. Here, we examined whether environmental samples can be used to support strategies of individual wild bird samplings. We validated a hollow-fiber ultrafiltration system to enrich AIV from surface water bodies. The results were compared to the analysis of corresponding sediments and environmentally deposited avian fecal samples.

Since the discovery of viruses, filtration for viral enrichment from liquid media has been important (32). However, these techniques were mostly designed to concentrate virus from cell culture supernatants or other smaller volumes such as clinical samples. Here, we used 10 L of surface water, which is easy to collect and ship to the laboratory by general services. Sample collection was mainly directed to smaller shallow water bodies within aquatic bird habitats. With respect to the enrichment effect, the Rexeed device proved to be the best method. The bacteriophage ϕ6 was indispensable as an internal control during validation runs and is useful also in the control of field samples with various loads of suspended sediment matter. An astonishingly high percentage of about 60% gave positive results for AIV-specific RNA by RT-qPCR. However, the virus load (*C_q_* range from 33.7 to 39.67) detected usually was close to the limit of detection and postfiltration methods failed to further enrich virus particles. The type of water sample (i.e., fresh, brackish, or salt) apparently had no influence on detectability (Table 2). Also, water samples obtained from water bodies at confirmed HPAI outbreak areas revealed only low virus RNA loads, comparable to previous studies (33). Several samples had to be examined after dilution due to the presence of PCR inhibitory substances. This severely restricted downstream analyses for sub- and pathotype determination. Consequently, only some samples could be further sub- or pathotyped. Other groups (e.g., reference 34) recommended using 2 M NaCl and 2 mM ethylenediamine tetra-acetic acid to minimize PCR inhibitors in difficult samples, but this has not been applied here to keep the number of steps and preparation time as low as possible. Also, AI virus isolation was generally unsuccessful although validation runs suggested that infectious AIV can be recovered qualitatively, and spiked infectious bacteriophage ϕ6 was regularly recovered from field samples (Fig. 3). Metagenomic sequencing approaches confirmed that the RNA recovered from the Rexeed columns is generally suitable for a large array of downstream investigations. However, low viral RNA loads again limited the success of further genotyping AIV since an additional myBaits-directed sequencing approach was required to enrich AIV nucleic acids nevertheless allowing at least sub- and pathotyping.

Given the very low virus loads recovered in the eluates, the use of larger sampling volumes might be appropriate. However, a substantial effect (gain of at least 1 log_10_ step of virus load) would require volumes >100 L. Since sending such volumes is difficult and costly, pumping surface water through the Rexeed device on a sampling spot would be required. Notwithstanding the fact that handling larger volumes requires increased technical support including a source of electricity in the field and skilled staff, we did not evaluate the suitability of Rexeed columns for such large sample volumes.

The success of detecting and characterizing AIV in surface water samples correlated with the initial virus load in the sampled water body. This was evident also by examining a sample from a 100-L water pool used for at least 2 days without changing the water by 10 HPAIV-infected mallards during an infection experiment. Virus detection and sub- and pathotyping were readily possible in the pooled sample.

Sediments of shallow water bodies had previously been successfully used to detect AIV RNA (35). Sedimentation of virus particles with other floating matter over time actually resembles a first enrichment step from water columns. Here, we examined 36 sediment samples matching with water samples from the same spot. In half of them, AIV RNA was detected but at the same low virus loads as in water samples. Nevertheless, subtyping attempts by RT-qPCRs revealed a larger spectrum of subtypes compared to the water column. This may reflect deposits of different AIV subtypes over time in sediments versus representation of more current AIV strains in the water column. In addition, matching qualitative results of corresponding water and sediment samples were limited indicating again that two different reservoirs of AI viruses are likely represented by these matrices. Although virus isolation failed for sediment samples, it cannot be excluded that AI viruses deposited in sediment retain infectivity and that sediments could serve as an environmental reservoir of infectivity. RNA extraction from sediments proved to be highly time consuming (at least 8 h) and not suitable for high-throughput analyses. The limited elution volume of about 30 μL generated by the method used here grossly restricts downstream diagnostic investigations.

With respect to the above-mentioned restrictions of using water and sediment samples for AIV surveillance, we included in our analysis environmentally deposited fresh avian fecal samples collected in the same region and during the same period. Although sampling was restricted to very few species including the barnacle goose, which was the main victim of the HPAIV wild bird epizootics since 2020 in Germany, only a minority (71/1705; 4.16%) of fecal samples from that species harbored AIV-specific RNA even during the peak of the epizootic, and only 10 samples could be determined as H5, of which just 3 were accessible to pathotyping as LP (*n* = 1) or HP-2.3.4.4b (*n* = 2), respectively. As with water and sediment samples, low viral RNA loads hampered downstream analyses. The presence of PCR inhibitory substances in the fecal matter likely contributed to the low viral loads detected. The use of different RNA extraction buffers claimed to reduce the inhibitory effects of fecal samples did not help to increase yields of viral RNA (not shown). In contrast, analysis of swabs collected from dead barnacle geese at the same time and from the same region revealed the presence of HPAIV H5Nx of clade 2.3.4.4b in 82.81% of carcasses analyzed. The limited usefulness of environmentally deposited avian feces for active AIV surveillance has been pointed out repeatedly (36, 37), and our current results confirm this. Several independent factors may contribute such as failure to collect sufficient fecal material, low cloacal excretion of AIV, and presence of inhibitory substances (porphyrins from chlorophyll, gut microbiota).

Despite over 100 years of practical work on virus purification via filtration devices, highly sensitive and technically undemanding methods for broad-spectrum viral surveillance applications are still scarce. Here, we confirmed previous reports that it is generally possible to detect AIV in environmental water and sediment samples. Yet, the viral loads are generally too low for virus isolation or further sub- and pathotyping purposes. Specific receptor-binding approaches such as those described in reference 38 have recently been found to be potentially more sensitive, also when smaller water volumes were investigated. Such techniques should be explored further. Alternative targets, such as environmentally deposited avian fecal samples, are laborious during collection and were shown to suffer from similar limitations as the water samples. Moreover, the spectrum of viruses detected by such active surveillance methods did not fully mirror an ongoing HPAIV epizootic among anseriform wild birds as detected by passive surveillance, which, in terms of sensitivity, remains unsurpassed.

## MATERIALS AND METHODS

### Use of bacteriophage ϕ6 as surrogate of influenza A viruses in environmental samples.

The bacteriophage ϕ6 of the *Cystoviridae* family resembles AIV by featuring a lipid envelope, a spherical shape of 75-nm diameter, and a segmented 13.4-kB RNA genome. However, in contrast to AIV, ϕ6 carries a double-stranded RNA genome. The bacteriophage ϕ6 replicates in a plant-specific bacterium and is considered a BSL-1 agent (39). These features render ϕ6 an interesting surrogate of influenza A viruses in validation experiments using environmental samples.

### (i) Bacterial media and buffers.

Medium 545 (tryptone soya broth [TSB]) was prepared as described by the DSMZ (German Collection of Microorganisms and Cell Cultures, Braunschweig, Germany) (40). The bouillon was supplemented with bacterial agar (Bact Agar Soldifying Agent; no. 214010; BD Diagnostics, East Rutherford, NJ, USA). For soft agar, 7.5 g per L broth was added and 15 g for solid agar. Phage buffer (49 mM Na_2_HPO_4,_ 22 mM KH_2_PO_4_, 86 mM NaCl, 1 mM MgSO_4_, and 1 mM CaCl_2_) was used for cultivation of bacteriophage ϕ6, as mentioned in reference 41. Salt-peptone buffer was used for dilution of ϕ6 in titration experiments (no. OXTV5016D; Oxoid Deutschland, Wesel, Germany).

### (ii) Culture of Pseudomonas syringae.

Pseudomonas syringae (DSZM-21482) was received from DSZM as a freeze-dried sample. For cultivating, the sample was mixed with 0.5 mL TSB liquid medium and 0.25 mL was plated and incubated overnight on TSB agar. A single bacterial colony was then transferred into 25 mL TSB bouillon and incubated for 20 h at 25°C and 222 rpm. Glycerine was then added to the bacterial suspension to a final concentration of 20% (vol/vol), and aliquots were stored at –80°C as a stock from which “working-aliquots” were grown as described.

### (iii) Culture of bacteriophage ϕ6.

The pseudomonas phage ϕ6 (DSM-21518) was obtained from DSZM. The bacteriophage was grown in cultures of its specific host bacterium Pseudomonas syringae. Generally, all culturing steps took place at 25°C for 20 h on an orbital shaker at 222 rpm. The bacteriophage ϕ6 was shipped on filter paper; half of the paper was placed onto a TSB-agar plate. In a reaction tube, 0.1 mL Pseudomonas syringae working solution (see below), 0.1 mL phage buffer, and 4 mL TSB-soft agar were mixed, poured over the paper on the plate, and incubated at 25°C. The next day, a single plaque colony was identified and transferred into 50 μL of phage buffer, mixed, and pipetted into 42 mL of a liquid culture of Pseudomonas syringae and incubated for 20 h at 25°C at 222 rpm. The bacteriophage-containing supernatant was recovered by centrifugation (5,594 × *g*, 30 min) and passed through a 0.45-μm filter. The filtrate was supplemented with glycerine to 20% (vol/vol), aliquoted, and stored at –80°C until further use.

### (iv) Titration of ϕ6 infectivity.

Classical plaque assays were used. A Pseudomonas syringae working stock (0.5 mL) was cultivated overnight in 300 mL TSB media, at 25°C and 222 rotations per minute (rpm). Then, 10 mL of the overnight bacterial suspension was diluted 1:5 in TSB media to start a fresh log-growth phase for 4 further hours. At an optical density (OD_600_) of 1.2 to 1.4, the suspension was ready to be used for the plaque assay. Phage suspensions were serially diluted 10-fold in salt-peptone buffer. Of each dilution step, 400 μL of the sample, 1,000 μL of hand-warm TSB soft agar, and 400 μL of Pseudomonas suspension (OD_600_ 1.2 to 1.4) were mixed. A total of 450 μL of this mixture were then pipetted into 1 well of a 6-well plate on top of 2.5 mL of solidified TSB agar. Triplicates of each dilution step and sample were used. A 10^−9^ ϕ6 dilution containing 19 (±7.4)/0.1 mL plaque-forming units (PFU) was used as a titrated positive control. As negative controls, salt-peptone buffer without ϕ6 and bacteria or bacteria only was used. After solidification of the soft agar layer, the plates were incubated upside down at 25°C for 22 h. Dilutions with ≤120 plaques were quantified with the naked eye, and PFU per volume were calculated.

### (v) ϕ6-Specific real-time quantitative RT-PCR.

Nine pairs (pairs 1 to 9) of primers were selected from conserved regions within the M-segment of ϕ6 and evaluated using a SYBR green RT-qPCR kit (SensiFAS SYBR No-ROX one-step kit; no. BIO-72001; Bioline Reagents Ltd., UK) at different annealing temperatures (50, 53, 56, and 60°C) in a three-step PCR-Program and at 60°C annealing for a two-step amplification program on a Bio-Rad Cfx1000 thermocycler (Bio-Rad Laboratories, Inc, Munich, Germany). Serial 10-fold dilutions of a stock of ϕ6 RNA were used as a template for validation runs, and PCR products were also checked by agarose gel electrophoresis.

For two primer pairs (pairs 3 and 5) yielding specific products at high sensitivity, FAM-labeled TaqMan probes were designed (Metabion International AG, Planegg, Germany, or IDT, Leuven, Belgium; Table S1). RT-qPCRs were assembled using the AgPath-ID one-step RT-PCR kit (Applied Biosystems; AM1005). Each 25 μL PCR contained 5 μL of ϕ6 RNA template. According to previous experiences with PCRs of viral double-stranded RNA (27), each RNA sample was subjected to an initial melting step (95°C for 5 min) whereafter the RNA was shock-cooled on a 96-well plate rack precooled in the −80° freezer before being added to the RT-qPCR mix. Cycling conditions on a CFX96 Real-Time-System C1000 Thermal Cycler (Bio-Rad, Munich, Germany) comprised of reverse transcription at 45°C for 10 min, *Taq* activation at 95°C for 10 min, followed by 43 cycles of 95°C for 15 s, 56°C for 20 s, and 72°C for 30 s.

### (vi) ϕ6-Specific T7-runoff trancripts.

A 95-bp-long ϕ6 fragment was chosen to generate a T7-runoff transcript as a positive control and to determine genome copy number. The fragment was ligated into the pCR 2.1-TOPO vector (K450002; Thermo Fisher Scientific, Waltham, MA, USA), and subsequently DH10B Competent Cells (EC0113; Thermo Fisher Scientific) were transformed with the plasmid. One hundred microliters of the transformation mixture was spread on an ampicillin-supplemented LB plate with IPTG and X-Gal for blue-white selection and incubated overnight. The next day, white bacterial colonies were picked and plasmids were prepared and sequenced with the M13-F and M13-RV primers. A plasmid harboring a fragment with an integer sequence inserted in the correct orientation was expanded, and plasmid DNA was recovered with the Qiagen Plasmid Midi kit (no. 12141; Qiagen, Hilden, Germany). Subsequently, 5 μg of the Midi prep plasmid DNA was linearized using KpnI (New England Biolabs, Ipswich, MA, USA). Linearized DNA was cleaned using the QIAquick Nucleotide Removal kit (no. 28306; Qiagen). For *in vitro* transcription, the RiboMAX Large Scale RNA Production System (no. P1300; Promega, Walldorf, Germany) was used. Priming was achieved with a T7 primer. After completion of the *in vitro* transcription, the preparation was digested using DNase Max (no. 15200-50; Qiagen). The RNA concentration was measured by Nanodrop. The genomic copy number of generated ϕ6 RNA runoff transcripts was calculated by using the http://endmemo.com/bio/dnacopynum.php calculator (42).

### Influenza A viruses.

AIV were obtained from the virus collection kept at the German National Reference Laboratory for Avian Influenza at Friedrich-Loeffler-Institut, Greifswald-Isle of Riems, Germany. Strain A/Greylag goose/Germany-MV/AR10080/2016 (H9N2) was grown in MDCK-II cells in a T25 cell-culture flask in the presence of TPCK-trypsin (2 μg/mL, final). Cultures were incubated for 48 h when extensive cytopathic effects had disrupted the cell monolayer. Following a freeze/thaw cycle the supernatant was clarified, aliquoted, and stored at –80°C.

**(i) Isolation and titration of influenza A viruses in cell culture and embryonated eggs.** Field samples to be analyzed for AIV infectivity were passed through a 0.45-μm filter (Millex-MCE 50S Filtereinheit, 45 μm; no. Millipore SLHA033SS; Merck KGaA, Darmstadt, Germany) to reduce bacterial contamination. A total of five 11-day-old embryonated specified pathogen-free-chicken eggs (ECE; VALO, Cuxhaven, Germany) were inoculated each with 0.2 mL of the filtered sample into the allantoic cavity. The eggs were incubated for up to 6 days at 37°C and candled daily for embryonic death. Eggs containing dead embryos, and all eggs at day 6 postinoculation, were chilled to 4°C for at least 16 h before allantois fluid (AAF) was harvested and tested for hemagglutination as described previously (43). In addition, RNA was extracted from selected AAFs and tested by influenza A virus generic RT-qPCR (see below). Infectivity titers were measured and calculated according to reference 44.

Furthermore, MDCK-II cell cultures (CRL-2936) were used for virus isolation, amplification, and titration of AIV infectivity in parallel. Methods have been outlined in reference 10.

**(ii) Influenza A virus-specific RT-qPCRs.** Extracted RNAs (see below) were tested for the presence of AIV by targeting in real-time RT-qPCR generically conserved regions of the M or NP genome segments (45, 46). An internal control (IC-2) as described in reference 47 was added to each sample during the RNA extraction process. Samples that showed an inhibition of the IC-2 amplification were reexamined at a 1:10 dilution. Samples testing positive for AIV-specific RNA were sub- and pathotyped by RT-qPCRs as described previously (48–51).

### Collection and processing of environmental samples.

Samples of 10 L of surface water were collected between November 2020 and October 2021 from various locations in Germany (Fig. 4). Characteristics of the sampling site and water type are presented in Table 4. The surface water was recovered and transported in 10-L canisters (no. 216-5333; VWR International GmbH, Darmstadt, Germany). To collect water, the canisters were dipped just below the water’s surface. Antarctic water samples were taken via pumping (Fig. S1). Water samples from Germany were either shipped in a Styrofoam box within 24 h after collection or immediately transferred to the lab, depending on the site. When accessible at the water sampling site, sediments (scooped out the sediment at the water extraction site) were collected as well. RNA from sediment samples was extracted as described in reference 52 and analyzed for AIV. The fecal samples were treated in the same way as those from active monitoring.

**TABLE 4:** Characteristics of water and sediment sampling locations

Sampling site ID^*a*^	Name	Longitude	Laditude	Water body	Water type^*b*^	Size (km^2^)	Maximum depth (m)^*c*^
1	Øland, Sweden	56.19505388	16.40021843	Baltic Sea, shallow inlet	Brackish	4	459 (inlet: 2 m)
2	Wadden Sea, Germany	54.52323015	8.863123177	North Sea, Wadden Sea	Salt	57,500	10
3	Wadden Sea, Germany	54.32637344	8.978028613	North Sea, Wadden Sea	Salt	57,500	10
4	Lake Duemmer, Germany	52.51297182	8.363835947	Lake	Fresh	14	1,4
5	Isle of Riems, Germany	54.18209948	13.35165043	Baltic Sea, shallow inlet	Brackish	514	14
6a	Isle of Koos, Germany, pond	54.17812491	13.40298791	Pond	Fresh	0,20	0,5
6b	Isle of Koos, Germany, beach area	54.16425804	13.41179628	Baltic Sea, shallow inlet	Brackish	514	14
7	Lake Max-Eyth, Germany	48.83135805	9.212351778	Lake	Fresh	0,17	2,3
8	Lake Constance, Germany	47.73414934	8.969084606	Lake, shallow inlet	Fresh	536	251 (inlet: 2 m)
A	Amt Röbel-Müritz, Germany	53,37700785	12.60598935	Fire pond	Fresh	100 m^2^	2
B	Amt Laage, Germany	53.92883034	12.34628184	Fire pond	Fresh	100 m^2^	1
C	County Greifswald, Germany	54.09577118	13.38036172	Southern Baltic Sea, shallow inlet 2	Brackish	514	14
D	Grimmen, Germany	54.1129066	13.04327085	Fire pond	Fresh	100 m^2^	2

a See Fig. 4 for depiction of site on map; A to D, samples originating from HPAI outbreak holdings; holding C is the site of a mallard sentinel station (60). The numbering corresponds with the sample IDs given in Table 2.

b Fresh, brackish, and salt (sea) water categorization is according to salt concentration: <1.000 ppm, 1.000 ≤35.000 ppm, and ≥35.000 ppm.

c All samples were taken in “rubber boots-depth” (50 cm) indicating sampling spots close to the riverbank.

A further 10-L sample originated from a water pool used in experimental AIV infection studies of mallards (10). This sample was taken on day 6 of the infection experiment, when the majority of mallards were excreting HPAIV of subtype H5N8, clade 2.3.4.4b, at high titers. During February 2021, ocean water samples were obtained by pumping from coastal areas of the Antarctic Weddell Sea as shown in Fig. S1 (sampling permission no. II 2.2-94033/176 in combination with no. II 2.8-94033/168). These samples were intended as a kind of negative control due to the expected high dilution effect.

**(i) Surface water filtration using the Vivaflow 200 device.** The Vivaflow 200 system utilizes tangential cross-flow filtration in combination with a cassette filter unit design. The additional equipment for the Vivaflow 200 filtration included Masterflex peristaltic pump (no. VFP001), Masterflex Easy Load pump head (size 15; no. VFA013), Tygon pumping tube (size, 15, 4.8 × 1.6 mm, 3 m once with Luer adapter; no. VFA003), and Y-connecting adapter (no. VFA005; all listed equipment was received from Sartorius Lab Instruments, Göttingen, Germany). The filtration was performed as described in the manual (53) with one exception; for the Vivaflow 200 setup, the fluid from the “rubbish” adapter was returned to the sampling bottle until the volume in the sampling bottle was reduced to a volume of 150 mL. Before and after each run, the cassettes were treated as described in the manual (53). The final concentrate consisted of 150 mL of which 50 mL were stabilized with 2% bovine serum albumin (albumin fraction V; no. 8076.4; Carl Roth, Karlsruhe, Germany) for further processing. The remaining 100 mL of the eluate were immediately frozen at –80°C.

**(ii) Surface water filtration using the Rexeed-25-A column.** Rexeed-25-A columns consist of a hollow fiber matrix. It is operated with a peristaltic pump (Hei-FLOW Precision 01; no. 224-1354; Heidolph Instruments GmbH & Co. KG, Schwabach, Germany). The filtration followed the principle of dead-end filtration, and the process took place as described in reference 54 with some changes. The sample streamed from the bottom red port through the column to the upper side port. After the complete sample was filtered, some leftovers of water were removed manually by removing the water from the side adapter of the filter with a syringe. The virus was eluted from the membrane by washing the membrane with either 0.2× PBS (no. 8418-12PCE; CHEMSOLUTE, Th. Geyer GmbH, Renningen, Germany) or 0.2× PBS supplemented with 0.001% Tween 80 (Tween 80; no. 655207-50; Merck, Darmstadt, Germany). The eluation took place from the upper (blue) to the bottom (red), up to a volume of 150 mL. The eluate of 150 mL was then treated as described for the VivaFlow concentrate (see above).

**(iii) Postfiltration measures.** SPE-DOM cartridge RNA extraction: Aliquots of 100 mL of an eluate obtained after Rexeed-25-A purification or a “raw” water sample were adjusted to pH 2 by using 37% smoking concentrated hydrochloric acid (Carl Roth, Karlsruhe, Germany) while stirring. The SPE-DOM column, with a volume of about 3 mL (Bond Elut PPL cartridge; no. 12105005; Agilent Technologies, Santa Clara, CA, USA), was preconditioned by adding a column volume of methanol (Carl Roth) before adding the sample for gravity- and pressure-assisted chromatography. The column then was washed twice with 0.01 MM HCl and dried for 5 min at room temperature. The eluate was obtained by rinsing the column using the same 0.5 mL methanol three times. To collect particulate matter, the filter of the column was removed, transferred to a 2-mL reaction tube containing 0.5 mL of cell culture medium, and homogenized (TissueLyser; Qiagen, Hilden, Germany) for 2 min at 300 Hz. After centrifugation, RNA was extracted from both the supernatant of the homogenized particulate matter and the column eluate using the QIAamp Viral RNA kit (no. 52904; Qiagen) as described in the manual.

For ultracentrifugation, aliquots of 100 mL of an eluate obtained after Rexeed-25-A purification or a “raw” water sample were ultracentrifuged at 175,000 × *g* for 3 h at 4°C in an SW-32-Ti Beckman rotor using Open-Top Thinwall Ultra-Clear Tubes (no. 344058; Beckman Coulter, Krefeld, Germany). The supernatant was decanted from the buckets, and pellets from three buckets were resuspended in a total volume of 0.5 mL 0.1× TE1 × TE buffer. RNA was extracted from resuspended pellets using the QIAamp Viral RNA minikit (no. 52904; Qiagen, Hilden, Germany) as described below.

The technical setup of the Rexeed filtration and postfiltration approaches is shown in Fig. 2.

**(iv) RNA extraction.** Rexeed-25-A and Vivaflow 200 eluates and other water samples, respectively, were processed using the NucliSens Magnetic Extraction kit (no. 200293; bioMérieux, Marcy-l’Étoile, France) as described previously (55). In brief, 5 mL of the sample was incubated for 10 min in 10 mL of lysis buffer (NUCLISENS easyMAG Lysis Buffer; no. 280134; bioMérieux, Marcy-l’Étoile, France). After removal of floating particles via centrifugation (5,889 × *g* for 5 min), the RNA extraction proceeded essentially as described in the manual.

In addition, the water enrichment Zymo Environ Water RNA kit (no. R2042; Zymo Research Europe GmbH, Freiburg, Germany) was utilized to determine the viral enrichment within the ultracentrifugation post-Rexeed-25-A filtration.

SPE-DOM eluates, ultracentrifugation pellets, feathers, and feces samples were extracted using the QIAamp Viral RNA (no. 52904; Qiagen, Hilden, Germany) system.

The RNA extraction of sediments from water bodies was performed via the RNeasy PowerSoil Total RNA kit (no. 12866-25; Qiagen, Hilden, Germany), according to the protocol provided by references 52.

**FIG 5:**
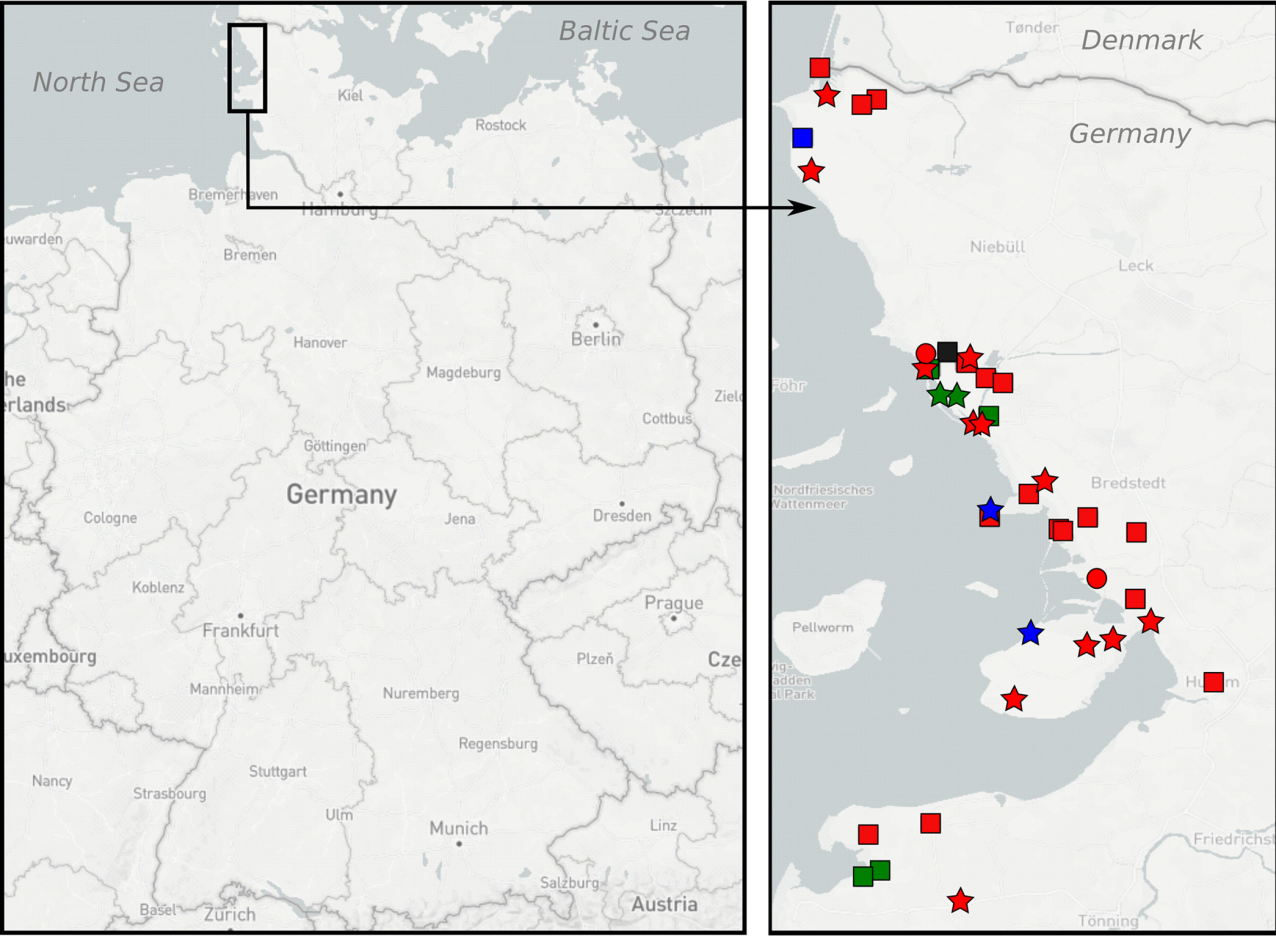
Geographic locations in the Wadden Sea national park in the German Federal State of Schleswig-Holstein depicting sampling sites of environmental fresh avian fecal samples of three anatid species (*Branta leucopsis*, red markings; *Branta bernicla*, blue markings; *Mareca penelope*, green markings) in 2020 (circles), 2021 (squares), and 2022 (stars).

**(v) Collection of environmental avian fecal samples (active surveillance).** During the winter months (November 2020 until March 2021 and December 2021 until April 2022), 1,620 and 500 avian fecal droppings were collected at locations (Fig. 5) in or adjacent to the German Wadden Sea national park. Samples were collected by ornithologically experienced rangers at sites where they observed large flocks of barnacle geese (Branta leucopsis), Eurasian wigeons (Mareca penelope), and brent geese (Branta bernicla) immediately before sampling fresh droppings. Several of the sampling sites were situated adjacent to surface water sampling spots (Fig. 5). For RNA extraction, approximately 100 mg of fecal matter was resuspended in 1 mL cell culture medium supplemented with penicillin/streptomycin (10,000 U/mL/10,000 μg/mL; no. A2213; Biochrom, Berlin, Germany) and shaken at room temperature at 230 rpm for 30 min (Varioshake VS 15 O; no. 9837945; LAUDA, Lauda-Königshofen, Germany). The medium was transferred to a 1.5-mL reaction tube and centrifuged for 5 min at 2,655 × *g* (Eppendorf centrifuge 5430R; no. 5428000205; Eppendorf SE, Hamburg, Germany) to remove larger floating particles. RNA extraction from the supernatant was performed using the NucleoMag VET kit (no. 744200.1; MACHEREY-NAGEL GmbH & Co. KG, Düren, Germany) on a KingFisher 96 BioSprint platform (Qiagen, Hilden, Germany).

**(vi) Metagenome sequencing of a field water sample.** A generic sequencing workflow as previously described in reference 56 was employed with some modifications. These included the use of the SuperScriptIV First-Strand cDNA Synthesis System (Invitrogen, Germany) as well as the NEBNext Ultra II Non-Directional RNA Second Strand Synthesis Module (New England Biolabs, Germany) to generate cDNA. Furthermore, the QIAseq Library Quant assay kit (Qiagen, Germany) was used for library quantification. A small part (5 μL) of the original sequencing library was sequenced using an Ion 530 chip in combination with the chemistry for 400-bp reads on an Ion Torrent S5XL instrument (Thermo Fisher Scientific, Germany). The remaining sequencing library (24.6 μL) was treated with a myBaits panel for avian viruses (including baits for Influenza) to specifically enrich virus nucleic acids as described (57) employing a hybridization time of 25 h at 64°C and sequenced as described.

The data sets were analyzed using the software RIEMS version 4 (58). For an in-depth analysis of AIV sequences, the Genome Sequencer software suite (version 2.6; Roche) was used to perform mapping analysis using A/Anas_crecca/Hubei/Chenhu1623_5/2014 (H5N6) and A/environment sample/China/TZ001/2021 (H5N8) as reference sequences. Obtained contigs were checked via blastn analysis (https://blast.ncbi.nlm.nih.gov/Blast.cgi).

### Data availability.

All data pertinent to this study are presented in tables and figures in the main text or in the supplemental materials.
