# Supplementary material for: Investigating Environmental Matrices for Use in Avian Influenza Virus Surveillance—Surface Water, Sediments, and Avian Fecal Samples
Source: Microbiol Spectr. 2023 Jan 26;11(2):e02664-22. doi: 10.1128/spectrum.02664-22 (PMC10100768; doi:10.1128/spectrum.02664-22)
Supplement: Supplemental file 1 — Fig. S1 and Tables S1 to S3. Download spectrum.02664-22-s0001.pdf, PDF file, 0.3 MB [file spectrum.02664-22-s0001.pdf]

Supplemental Material

Supplemental Figure 1: Geographic location of the Antarctic ocean water samples.

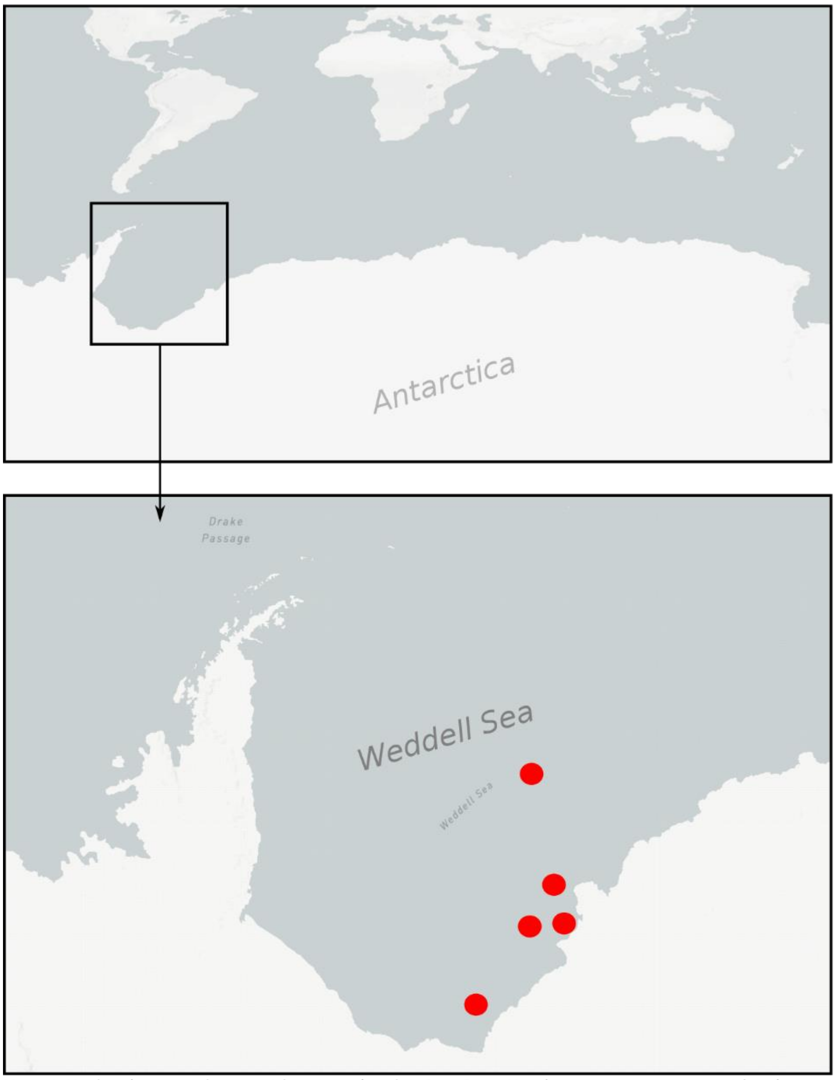

Maps © Mapbox (www.mapbox.com/about/maps) and © OpenStreetMap (www.openstreetmap.org/about)

Supplemental Table 1: Primer and probe sequences used in PCR amplification of fragments of the bacteriophage ϕ6 M gene.

Mix	Orientation	Sequence 5' → 3'
1	Fw	GGTTGGCCAAGCGCGAT
	Rev	TTT AGC CCC GAT GTG CAC A
2	Fw	ATG CCA CTC GTG CCA AGCA
	Rev	CAC AGA TCG AGG AGT GGAA
	Probe	ACC TGT GGG TTG GCG ATA ACG TTG T
3	Fw	CCC ACG TAA GCT CGT CGA
	Rev 1	GAG AGG ATG CCA CCA ACG A
	Rev 2	GAGAGGTTGCCTCCAACGA
	Probe	FAM-CCT GAT GGT GTA CGC TCG CGA TC-BHQ1
4	Fw	CAG ATC ATC GAA GCG GAC A
	Rev	CTG GTG CCA CAA CGT CTC
5	Fw	ACT GCG GAC TCC AAC GGT
	Rev	AGC GGC ATT GAC CTG CTC
	Probe	FAM-CGT GGA CGA CGT GCG TAA CTA-BHQ1
6	Fw	GGC ACC TTG GCA GTC GGT
	Rev	CCA CTG GTG CCG TGG ACA
7	Fw	GCCT TGA CCT GGC CAC CA
	Rev	CAA GGC GCA GCC CAT GCA
8	Fw	CCA CTC GTG CCA AGC AGA
	Rev	GTT CCA CTC CTC GAT CTG TG
9	Fw	CAT TCG TTC CCT CGA GTT GA
	Rev	CAG ACC GAC CAA CGC GTA

Supplemental Table 2: Details of validation runs of two ultrafiltration approaches.

	Parameter	Vivaflow		Rexeed-PBS								Rexeed-PBST			
ϕ6		1	2	1	2	3	4	5	6	7	8	1	2	3	4
10L	Cq	31.38	32.58	31.79	31.19	32.06	32.20	30.81	33.5	22.85	24.06	31.62	30.78	31.75	32.71
150 mL	Cq	27.72	27.89	24.47	26.01	25.23	25.44	23.61	29.42	15.93	17.82	25.73	25.22	25.32	26.35
Delta Cq	Cq	3.67	4.69	7.31	5.19	6.83	6.76	7.2	4.08	6.92	6.24	5.89	5.56	6.43	6.36
10 L	RNA copies	2.75x10^{4}	1.06x10^{4}	2.00x10^{4}	3.20x10^{4}	1.60x10^{4}	1.43x10^{4}	4.34x10^{4}	5.13x10^{3}	2.40x10^{7}	9.18x10^{6}	2.28x10^{4}	4.43x10^{4}	2.05x10^{4}	9.58x10^{3}
150 mL	RNA copies	5.05x10^{5}	4.41x10^{5}	6.61x10^{6}	1.95x10^{6}	3.62x10^{6}	3.06x10^{6}	1.31x10^{7}	1.31x10^{5}	5.80x10^{9}	1.30x10^{9}	2.44x10^{6}	3.65x10^{6}	3.36x10^{6}	1.49x10^{6}
Factor	n	1.83x10^{1}	4.14x10^{1}	3.31x10^{2}	6.12x10^{1}	2.25x10^{2}	2.13x10^{2}	3.02x10^{2}	2.54x10^{1}	2.42x10^{2}	1.41x10^{2}	1.07x10^{2}	8.24x10^{1}	1.64x10^{2}	1.55x10^{2}
10 L	Pfu	7.08x10^{4}	3.47x10^{5}	7.73x10^{5}	8.53x10^{5}	9.85x10^{5}	9.58x10^{7}	1.67x10^{5}	8.89x10^{7}	5.00x10^{7}	8.65x10^{7}	9,81E+05	7.79x10^{5}	8.97x10^{5}	9.67x10^{5}
150 mL	Pfu	1.78x10^{3}	3.00x10^{1}	1.23x10^{7}	9.82x10^{6}	2.23x10^{7}	1.45x10^{9}	3.94x10^{7}	2.97x10^{9}	1.43x10^{9}	6.97x10^{8}	1,19E+07	1.70x10^{7}	1.25x10^{7}	1.12x10^{7}
Factor	n	2.52x10^{-2}	8.65x10^{-5}	1.59x10^{1}	1.15x10^{1}	2.26x10^{1}	1.51x10^{1}	2.36x10^{2}	3.34x10^{1}	2.86x10^{1}	8.06x10^{0}	1,22E+01	2.18x10^{1}	1.39x10^{1}	1.16x10^{1}
**H9N2**															
10L	Cq	30.47	31.34	27.76	28.54	29.32	32.35	33.57	30.84	31.97	33.57	30.01	30.08	29.32	35.32
150 mL	Cq	27.05	28.18	22.57	22.67	23.33	26.8	27.08	26.54	27.14	28.18	24.84	25.56	26.5	27.41
Delta Cq		3.42	3.16	5.19	5.87	5.99	5.55	6.49	4.3	4.83	5.39	5.17	4.52	2.82	7.91
Virus Isolation	n/n	1	1	1	1	1	1					1	1	1	1

Supplemental Table 3: Technical comparison of two ultrafiltration approaches validated here.

Parameter	VivaFlow	Rexeed
Filtration principle	Tangential cross flow	Dead-end
Filter unit design	Cassette	Hollow fibre bundles
Reusability	Yes; 5-10 times	No; single use
Orginally designed for	Purifying pharmaceutical products	Medical blood dialysis
Approx. costs in €/run	64, filters re-used 10 times	80
Approx. running time	>1hour	40 minutes
Total running (filter preparation, filtration, eluation, filter cleaning)	>5 hours	~ 1 hour
